# Perioperative Body-Composition and Phase-Angle Measures in Women Undergoing Breast Cancer Surgery After Neoadjuvant Chemotherapy or Treated Through a Surgery-First Pathway (BC-NUTRITION): Exploratory 2-Cohort Pilot Analysis

**DOI:** 10.2196/102885

**Published:** 2026-09-30

**Authors:** Yu Liu, Congyi Ma, Guanfeng Chen, WangJing Hu, Lu Li, Jingxia Pan, Huan Li

**Affiliations:** 1 Third Affiliated Hospital of Sun Yat-sen University, No. 600, Tianhe Road, Tianhe District, Guangzhou, Guangdong, 510630, China, 86 13570400112

**Keywords:** breast cancer, neoadjuvant chemotherapy, body composition, phase angle, bioelectrical impedance analysis

## Abstract

**Background:**

Breast cancer survivorship requires attention to treatment-related changes in body composition, nutritional status, and quality of life. Bioelectrical impedance phase angle is influenced by tissue electrical properties and fluid distribution and may provide complementary whole-body and segmental information.

**Objective:**

This study described perioperative body-composition and phase-angle measures in women undergoing breast cancer surgery after neoadjuvant chemotherapy or treated through a surgery-first pathway. It also estimated T0-to-T2 changes and documented the availability of selected longitudinal variables.

**Methods:**

BC-NUTRITION (Body Composition and Nutrition Trajectories in Breast Cancer) is a prospective 2-cohort observational study with 5 planned assessments from admission to 6 months after discharge. The present exploratory pilot analysis included 30 women in cohort A, assessed after neoadjuvant chemotherapy, and 65 women in cohort B, who were treated through a surgery-first pathway. T1 measurements were displayed descriptively; inferential analyses used T0 values and participant-level T0-to-T2 changes. T0 between-cohort comparisons used Welch 2-sample *t* tests, within-cohort changes used paired *t* tests, and between-cohort differences in change used Welch tests. Benjamini-Hochberg *q* values were calculated across the 13 between-cohort change comparisons.

**Results:**

All 95 participants had complete data for the 13 selected longitudinal variables at T0, T1, and T2. At T0, cohort A had lower albumin, absolute lymphocyte count, prognostic nutritional index, and whole-body 50-kHz phase angle. The whole-body phase-angle means were 4.64° (SD 0.66) and 5.23° (SD 0.62), with a difference of −0.59° (95% CI −0.88° to −0.31°; *P*<.001). Cohort A had a higher whole-body extracellular water to total body water ratio (ECW/TBW) than cohort B (0.3890 vs 0.3815; *P*<.001). Ipsilateral upper-limb 50-kHz phase angle changed by −0.50° in cohort A and −0.51° in cohort B between T0 and T2. The between-cohort difference was 0.01° (95% CI −0.17° to 0.19°; *P*=.93). The corresponding difference in whole-body phase-angle change was 0.14° (95% CI −0.02° to 0.30°; *P*=.09). No *q* value for the 13 between-cohort change comparisons was below 0.05.

**Conclusions:**

The treatment pathways differed in selected laboratory and whole-body bioelectrical measures at T0. The mean upper-limb phase angle ipsilateral to the recorded tumor side decreased in both cohorts between T0 and T2. All five prespecified perioperative feasibility thresholds were met in the available T0-to-T2 data. Assessment acceptability and T3/T4 follow-up were not evaluated. Definitive interpretation requires standardized acquisition, a prechemotherapy baseline, relevant clinical covariates, and outpatient follow-up.

## Introduction

Breast cancer is the most commonly diagnosed cancer among women and is the leading cause of cancer death among women in 112 countries [1]. Among women diagnosed from 2010 to 2014, age-standardized 5-year net survival reached at least 85% in 25 countries [2]. As survival improves, attention increasingly extends beyond oncological control to posttreatment health. Relevant outcomes include body composition, persistent fatigue, cardiometabolic morbidity, and patient-reported quality of life [3-5].

Body composition is increasingly recognized as a clinically relevant and potentially modifiable phenotype across the cancer-care continuum. Skeletal muscle supports physical function and metabolic homeostasis, whereas excess or ectopic adiposity may promote inflammatory and metabolic stress. In nonmetastatic breast cancer, lower muscle mass and greater adiposity have been associated with shorter survival [6]. Greater visceral or intramuscular adiposity has also been associated with lower relative dose intensity during anthracycline- and taxane-based chemotherapy [7]. A systematic review linked sarcopenia to shorter survival and greater chemotherapy toxicity [8]. Higher visceral adiposity has also been associated with worse distant disease-free survival after neoadjuvant chemotherapy [9]. These observations support attention to exercise, nutrition, and weight management during treatment. Evidence for specific interventions and their optimal timing remains incomplete [4,5].

At 50 kHz, the phase angle is calculated as arctan(reactance/resistance) × 180/π [10]. It expresses the relationship between the capacitive and conductive components of bioelectrical impedance. The phase angle is influenced by tissue composition and hydration. It is often interpreted as an indirect index of cell-membrane function and body cell mass, rather than a direct measure of cellular health. A systematic review in breast cancer found substantial variation in phase-angle values and methods, with inconsistent evidence for survival outcomes [11]. In advanced cancer, lower phase angle has been associated with nutritional status and survival. However, study populations, measurement procedures, and proposed cut-offs have varied [12]. Whole-body and segmental phase angle are therefore related but anatomically distinct measures. Upper-limb bioimpedance has been studied for breast cancer−related lymphedema [13,14]. Those studies evaluated limb impedance or interlimb ratios and did not validate short-term segmental phase-angle change as a marker of lymphatic injury.

Two clinically important questions remain. First, do women presenting for surgery after neoadjuvant chemotherapy differ in perioperative body-composition and phase-angle measures from women treated through a surgery-first pathway? Existing studies have described body-composition changes during neoadjuvant chemotherapy or associations between pretreatment body composition and outcomes, but they did not report a contemporaneous comparison of perioperative change between postneoadjuvant and surgery-first cohorts [15-17]. Because cohort A in the present study had no prechemotherapy bioimpedance assessment, the design cannot estimate the change caused by neoadjuvant chemotherapy. Second, can perioperative measurements be extended into reliable longer-term nutritional and body-composition trajectories? BC-NUTRITION (Body Composition and Nutrition Trajectories in Breast Cancer) was designed as a prospective 2-cohort study with 5 assessments from admission to 6 months after discharge. The current exploratory analysis included 95 participants assessed at T0, T1, and T2. Its objectives were to describe perioperative body-composition and phase-angle measures, estimate T0-to-T2 changes, evaluate the prespecified perioperative feasibility thresholds, and identify outcomes requiring later follow-up.

## Methods

### Study Design

BC-NUTRITION is a prospective, 2-cohort observational study conducted at The Third Affiliated Hospital of Sun Yat-sen University in Guangzhou, China. The study planned a 50-participant preliminary review within a parent-study target of 214 participants. Here, “pilot” denotes a preliminary, hypothesis-generating analysis of the 95 participants with perioperative assessments. Five prespecified perioperative feasibility thresholds were assessed. They covered preliminary sample size, T0-to-T2 retention, completeness of the T2 primary outcome, baseline balance across 15 indices, and loss to follow-up before T2. The current analysis covered assessments at T0, T1, and T2. Reporting followed the Strengthening the Reporting of Observational Studies in Epidemiology statement [18].

### Participants

Eligible participants were women aged 18 to 80 years with pathologically confirmed primary breast cancer and planned adjuvant or neoadjuvant chemotherapy. Participants also needed the capacity to consent, the ability to use a smartphone, and a willingness to participate. Exclusion criteria included diabetes mellitus and severe cardiac, pulmonary, hepatic, or renal disease. Other exclusions were a second primary malignancy and conditions expected to alter hydration, cause edema, or affect muscle mass or function. Concurrent enrollment in another intervention, treatment outside of curative intent, and use of medical nutrition products outside the study procedures were also exclusion criteria.

### Cohort Allocation and Treatment

Treatment pathway was determined by the attending breast cancer team in accordance with national guidelines [19]. Breast-conserving surgery was considered when clinically appropriate. Allocation was observational rather than randomized. Cohort A included 30 women who received neoadjuvant chemotherapy before surgery. Cohort B included 65 women treated through a surgery-first pathway. The observed 30-to-65 distribution was not a planned allocation ratio. Recorded breast procedures were modified radical mastectomy, breast-conserving surgery, and total mastectomy. Axillary procedure and extent were not captured separately, so axillary management was not analyzed. Chemotherapy regimen, cumulative exposure, and treatment toxicity were outside the present analysis. Between-cohort comparisons are therefore descriptive.

### Measurements and Assessment Schedule

Five assessments were scheduled per participant (Figure 1; Table 1). T0 was admission before surgery, T1 was on postoperative day 1 after mobilization, and T2 was on postoperative day 7. T3 and T4 were scheduled at 1 and 6 months after discharge. Bioelectrical impedance analysis (BIA) was scheduled at T0, T1, and T2. The current dataset contained whole-body and segmental bioimpedance measurements at 5 and 50 kHz. It also included drain duration and total postoperative drainage volume. Nutritional Risk Screening 2002 (NRS-2002), Patient-Generated Subjective Global Assessment (PG-SGA), Patient Health Questionnaire-9 (PHQ-9), and Functional Assessment of Cancer Therapy-Breast (FACT-B) were planned [20-23]. These measures and T3/T4 follow-up were not included in the current analysis.

**Table 1.** Selected BC-NUTRITION (Body Composition and Nutrition Trajectories in Breast Cancer) assessment schedule^a^.

Measurement	T0 admission	T1 postoperative day 1	T2 postoperative day 7	T3 1 month postdischarge	T4 6 months postdischarge
Demographics and disease information	✓				
InBody bioelectrical impedance	✓	✓	✓		
NRS-2002^b^	✓	✓	✓	✓	✓
PG-SGA^c^	✓	✓	✓	✓	✓
PHQ-9^d^		✓	✓	✓	✓
FACT-B^e^	✓			✓	✓
Upper-limb edema assessment	✓	✓	✓	✓	✓
Standing height	✓				
Body weight	✓	✓	✓	✓	✓
Waist and hip circumferences	✓	✓	✓	✓	✓

a T0 was at admission before surgery, T1 was postoperative day 1 after mobilization, and T2 was postoperative day 7. T3 and T4 were scheduled at 1 and 6 months after discharge. The current analysis included T0, T1, and T2.

b NRS-2002: Nutritional Risk Screening 2002.

c PG-SGA: Patient-Generated Subjective Global Assessment.

d PHQ-9: 9-item Patient Health Questionnaire.

e FACT-B: Functional Assessment of Cancer Therapy-Breast.

BIA was performed with an InBody 770 multifrequency direct segmental analyzer in a dedicated room between 06:00 and 09:00. Participants were fasting and wore light clothing. They voided the bladder, rested supine for at least 10 minutes, removed metal jewelry, and then completed the measurement barefoot in the standard standing position. A trained breast-care nurse confirmed contact with the 8 tactile hand and foot electrodes and instructed participants to remain still with their arms separated from the trunk during the measurement. The analyzer measures the right arm, left arm, trunk, right leg, and left leg at multiple frequencies. The present analysis used the 5-kHz and 50-kHz measurements. BIA was the study measurement method, and no reference body-composition method was available. The outputs are therefore reported as device-derived exploratory measures. Baseline laboratory data included aspartate aminotransferase, alanine aminotransferase, total protein, albumin, and absolute lymphocyte count. Prognostic nutritional index was calculated as albumin in g/L plus 5 times the absolute lymphocyte count in 10⁹/L [24].

### Outcomes

The parent protocol defined longitudinal body composition as the primary outcome. The 13-variable outcome set and main contrast reported here were treated as exploratory. Whole-body outcomes comprised weight, BMI, skeletal muscle mass, fat-free mass, body fat mass, percent body fat, and visceral fat area. They also included device-reported skeletal muscle index, extracellular water to total body water ratio (ECW/TBW), and 50-kHz phase angle. Segmental outcomes comprised 5-kHz reactance, 50-kHz reactance, and 50-kHz phase angle for the ipsilateral upper limb. Baseline values and participant-level T0-to-T2 changes were analyzed. T1 measurements were displayed descriptively and were excluded from inferential comparisons. Whole-body and ipsilateral phase angle were analyzed separately. The principal contrast was the between-cohort difference in mean change.

### Sample Size and Parent-Study Context

The parent-study sample-size calculation used Power Analysis and Sample Size (PASS; version 15) and assumed equal cohort sizes, a standardized mean difference of 0.50, a 2-sided α of .05, and 90% power. It yielded 85 participants per cohort before allowance for attrition and 107 per cohort after a 20% allowance, giving a target of 214. A preliminary review was planned after at least 50 participants. The present report analyzes all 95 participants, including 30 in cohort A and 65 in cohort B. This distribution was observational and was not a planned 1-to-2 allocation. The perioperative SDs reported below provide preliminary variance estimates. An end point-specific recalculation would additionally require a clinically important difference and the within-participant correlation structure. No revised sample-size calculation was performed for this report.

### Statistical Analysis

Continuous variables were summarized as mean (SD), and categorical variables as n (%). Between-cohort comparisons of T0 continuous variables used Welch 2-sample *t* tests. Tumor laterality used a 2 × 2 Pearson *χ*^2^ test, and procedure type used an omnibus 2 × 3 Pearson *χ*^2^ test. Within-cohort T0-to-T2 changes were assessed with paired *t* tests. Between-cohort differences in mean change were assessed with Welch 2-sample *t* tests on participant-level change scores, with 95% CIs based on the Welch-Satterthwaite approximation. All *P* values were 2-sided.

For the 13 between-cohort change comparisons, Benjamini-Hochberg-adjusted *P* values are reported as *q* values. Adjustment was performed within this 13-comparison family. The 26 within-cohort comparisons were descriptive exploratory analyses and were not included in that family. The main analysis retained all 95 participants. A post hoc sensitivity analysis omitted 2 participants identified by post hoc data-quality flags. One flag identified a T2 BMI discrepancy greater than 3 kg/m². It compared recorded BMI with a value recalculated from T2 weight and height inferred from T0 measurements. The second flag identified an absolute T0-to-T2 weight change greater than 10 kg. These flags were not prespecified exclusion criteria. They did not justify changing any recorded measurement without review of the original measurement records.

All 95 participants had complete data for the 13 reported variables at T0, T1, and T2. InBody Score was not analyzed and was missing for 2 participants at T0, 5 at T1, and 2 at T2. No values were imputed. T3 and T4 were outside the current analysis and were not treated as missing within the T0-to-T2 analysis. Analyses were conducted with Python 3.13.9, pandas 2.3.3, NumPy 2.3.5, and SciPy 1.16.3. The completeness of the perioperative data and the post hoc sensitivity analyses are provided in Multimedia Appendix 1.

### Ethical Considerations

The Medical Ethics Committee of The Third Affiliated Hospital of Sun Yat-sen University granted initial approval on July 16, 2024 (II2024-199-01), for the period from July 16, 2024, through July 15, 2025. The committee issued the first continuing-review approval on June 20, 2025 (II2024-199-02), for July 16, 2025, through July 15, 2026. It issued the second continuing-review approval on July 15, 2026 (II2024-199-03), for July 16, 2026, through July 15, 2027. Together, these 3 consecutive approval periods provided uninterrupted ethics coverage from July 16, 2024, through July 15, 2027, with no gap. The present analysis used the May 27, 2026, data cut. All participant recruitment and every T0, T1, and T2 assessment included in this data cut occurred within one of these approved periods. Written informed consent was required before study procedures. Participant-level analysis files used sequential study identifiers and excluded names, hospital registration numbers, exact dates, and free-text identifiers.

## Results

### Analysis Population

The analysis included 95 participants, with 30 in cohort A and 65 in cohort B (Figure 1). All participants had complete data for the 13 variables in Tables 2 and 3 at T0, T1, and T2. Procedure type and all other variables in Table 4 were complete. The baseline, procedure-type, and T0-to-T2 analyses included all 95 participants.

**Table 2.** Within-cohort changes in body-composition and bioimpedance measures between T0 and T2^a^.

Variable and cohort	T0 mean (SD)	T2 mean (SD)	Change (95% CI)	Paired *t* test (*df*)	*P* value
Body weight (kg)					
A (n=30)	59.41 (9.34)	59.27 (9.07)	−0.14 (−0.43 to +0.15)	−1.02 (29)	.32
B (n=65)	59.42 (10.58)	59.19 (10.09)	−0.23 (−0.75 to +0.28)	−0.90 (64)	.37
BMI (kg/m²)					
A (n=30)	24.13 (3.33)	24.24 (3.06)	+0.11 (−0.28 to +0.50)	+0.58 (29)	.57
B (n=65)	24.06 (3.70)	23.98 (3.46)	−0.08 (−0.29 to +0.13)	−0.75 (64)	.46
Skeletal muscle mass (kg)					
A (n=30)	20.94 (2.64)	20.83 (2.39)	−0.11 (−0.38 to +0.16)	−0.86 (29)	.40
B (n=65)	21.23 (3.10)	21.22 (3.11)	−0.02 (−0.20 to +0.17)	−0.18 (64)	.86
Fat-free mass (kg)					
A (n=30)	39.17 (4.41)	39.03 (4.00)	−0.14 (−0.60 to +0.31)	−0.64 (29)	.53
B (n=65)	39.30 (5.21)	39.38 (5.17)	+0.08 (−0.26 to +0.43)	+0.49 (64)	.62
Body fat mass (kg)					
A (n=30)	20.24 (5.91)	20.64 (5.75)	+0.40 (−0.56 to +1.37)	+0.85 (29)	.40
B (n=65)	20.13 (7.30)	19.81 (7.01)	−0.32 (−0.73 to +0.10)	−1.54 (64)	.13
Percent body fat (%)					
A (n=30)	33.51 (5.34)	34.10 (5.11)	+0.59 (−0.76 to +1.95)	+0.90 (29)	.38
B (n=65)	33.04 (7.27)	32.71 (7.25)	−0.33 (−0.83 to +0.16)	−1.35 (64)	.18
Visceral fat area (cm²)					
A (n=30)	101.67 (33.42)	104.74 (32.53)	+3.07 (−2.91 to +9.04)	+1.05 (29)	.30
B (n=65)	98.50 (45.09)	97.78 (42.54)	−0.72 (−3.38 to +1.94)	−0.54 (64)	.59
Device-reported skeletal muscle index (kg/m²)					
A (n=30)	6.17 (0.74)	6.05 (0.66)	−0.13 (−0.22 to −0.04)	−2.87 (29)	.008
B (n=65)	6.20 (0.68)	6.16 (0.64)	−0.04 (−0.10 to +0.02)	−1.46 (64)	.15
Ipsilateral upper-limb 5 kHz reactance (Ω)					
A (n=30)	13.47 (3.42)	10.41 (2.62)	−3.05 (−4.01 to −2.09)	−6.51 (29)	<.001
B (n=65)	16.38 (2.90)	12.60 (2.62)	−3.78 (−4.49 to −3.06)	−10.50 (64)	<.001
Ipsilateral upper-limb 50 kHz reactance (Ω)					
A (n=30)	25.70 (4.60)	22.44 (4.15)	−3.26 (−4.62 to −1.90)	−4.91 (29)	<.001
B (n=65)	30.21 (4.46)	26.41 (4.42)	−3.80 (−4.69 to −2.91)	−8.54 (64)	<.001
Ipsilateral upper-limb 50-kHz phase angle (°)					
A (n=30)	4.18 (0.63)	3.68 (0.64)	−0.50 (−0.66 to −0.34)	−6.60 (29)	<.001
B (n=65)	4.71 (0.64)	4.20 (0.65)	−0.51 (−0.60 to −0.42)	−11.09 (64)	<.001
Whole-body 50-kHz phase angle (°)					
A (n=30)	4.64 (0.66)	4.53 (0.65)	−0.11 (−0.25 to +0.03)	−1.64 (29)	.11
B (n=65)	5.23 (0.62)	4.98 (0.63)	−0.25 (−0.33 to −0.16)	−5.79 (64)	<.001
Whole-body ECW/TBW^b^					
A (n=30)	0.3890 (0.0064)	0.3889 (0.0066)	−0.0001 (−0.0018 to +0.0016)	−0.12 (29)	.90
B (n=65)	0.3815 (0.0072)	0.3839 (0.0071)	+0.0023 (+0.0011 to +0.0035)	+3.80 (64)	<.001

a Values are participant-level means and mean T2 minus T0 changes with 95% CIs. Two-sided paired *t* tests included 30 participants in cohort A and 65 in cohort B. The ipsilateral upper limb was selected according to recorded tumor laterality. The 26 *P* values are unadjusted exploratory results outside the 13-comparison family in Table 3. Device-reported skeletal muscle index was not treated as equivalent to computed tomography–derived skeletal muscle index because the device algorithm was unavailable.

b ECW/TBW: extracellular water to total body water ratio.

**Table 3.** Between-cohort differences in mean change between T0 and T2^a^.

Variable	Cohort A change (95% CI)	Cohort B change (95% CI)	Cohort A minus cohort B difference (95% CI)	*P* value	*q* value
Body weight (kg)	−0.14 (−0.43 to +0.15)	−0.23 (−0.75 to +0.28)	+0.09 (−0.50 to +0.68)	.76	.823
BMI (kg/m²)	+0.11 (−0.28 to +0.50)	−0.08 (−0.29 to +0.13)	+0.19 (−0.25 to +0.63)	.39	.607
Skeletal muscle mass (kg)	−0.11 (−0.38 to +0.16)	−0.02 (−0.20 to +0.17)	−0.10 (−0.42 to +0.23)	.55	.652
Fat-free mass (kg)	−0.14 (−0.60 to +0.31)	+0.08 (−0.26 to +0.43)	−0.23 (−0.79 to +0.33)	.42	.607
Body fat mass (kg)	+0.40 (−0.56 to +1.37)	−0.32 (−0.73 to +0.10)	+0.72 (−0.32 to +1.76)	.17	.455
Percent body fat (%)	+0.59 (−0.76 to +1.95)	−0.33 (−0.83 to +0.16)	+0.93 (−0.50 to +2.36)	.20	.455
Visceral fat area (cm²)	+3.07 (−2.91 to +9.04)	−0.72 (−3.38 to +1.94)	+3.79 (−2.69 to +10.26)	.25	.455
Device-reported skeletal muscle index (kg/m²)	−0.13 (−0.22 to −0.04)	−0.04 (−0.10 to +0.02)	−0.08 (−0.19 to +0.02)	.12	.455
Ipsilateral upper-limb 5-kHz reactance (Ω)	−3.05 (−4.01 to −2.09)	−3.78 (−4.49 to −3.06)	+0.72 (−0.46 to +1.90)	.23	.455
Ipsilateral upper-limb 50-kHz reactance (Ω)	−3.26 (−4.62 to −1.90)	−3.80 (−4.69 to −2.91)	+0.54 (−1.06 to +2.14)	.50	.652
Ipsilateral upper-limb 50-kHz phase angle (°)	−0.50 (−0.66 to −0.34)	−0.51 (−0.60 to −0.42)	+0.01 (−0.17 to +0.19)	.93	.931
Whole-body 50-kHz phase angle (°)	−0.11 (−0.25 to +0.03)	−0.25 (−0.33 to −0.16)	+0.14 (−0.02 to +0.30)	.09	.455
Whole-body ECW/TBW^b^	−0.0001 (−0.0018 to +0.0016)	+0.0023 (+0.0011 to +0.0035)	−0.0024 (−0.0045 to −0.0004)	.02	.268

a Changes were calculated for each participant as T2 minus T0. Cohort A included 30 participants and cohort B included 65. Cohort-specific CIs used the 1-sample t distribution. Differences between cohorts and their 95% CIs used 2-sided Welch *t* tests with the Welch-Satterthwaite approximation. The *q* values are Benjamini-Hochberg-adjusted *P* values across the 13 comparisons. Estimates were calculated from unrounded participant-level values. *P* values across the 13 comparisons. Estimates were calculated from unrounded participant-level values. Device-reported skeletal muscle index was not treated as equivalent to computed tomography–derived skeletal muscle index.

b ECW/TBW: extracellular water to total body water ratio.

**Table 4.** Participant characteristics and perioperative measures in the pilot data set (n=95)^a^.

Characteristic	Cohort A (NAC^b^) (n=30)	Cohort B (surgery first) (n=65)	Test statistic (*df*)	*P* value
Age (y), mean (SD)	51.57 (8.28)	52.03 (12.65)	−0.21^c^ (82.0)	.83
Tumor left-sided, n (%)	19 (63.33)	38 (58.46)	0.20^d^ (1)	.65
Modified radical mastectomy, n (%)	22 (73.33)	22 (33.85)	13.76^d^ (2)	.001
Breast-conserving surgery	5 (16.67)	17 (26.15)		
Total mastectomy	3 (10.00)	26 (40.00)		
Aspartate aminotransferase (IU/L), mean (SD)	25.03 (7.24)	19.91 (6.29)	3.34^c^ (50.0)	.002
Alanine aminotransferase (IU/L), mean (SD)	26.60 (11.11)	18.42 (10.52)	3.39^c^ (53.8)	.001
Total protein (g/L), mean (SD)	70.69 (4.64)	73.78 (4.92)	−2.96^c^ (59.6)	.004
Albumin (g/L), mean (SD)	40.52 (4.10)	42.58 (3.21)	−2.42^c^ (46.1)	.020
Absolute lymphocyte count (×10⁹/L), mean (SD)	1.17 (0.41)	1.79 (0.58)	−6.03^c^ (77.4)	<.001
Prognostic nutritional index, mean (SD)	46.36 (5.06)	51.55 (3.97)	−4.96^c^ (46.1)	<.001
Duration of drain placement (d), mean (SD)	6.70 (1.68)	5.55 (2.90)	2.42^c^ (88.1)	.02
Total postoperative drainage volume (mL), mean (SD)	433.87 (210.04)	358.34 (267.98)	1.49^c^ (70.8)	.14
Body weight (kg), mean (SD)	59.41 (9.34)	59.42 (10.58)	−0.01^c^ (63.4)	>.99
BMI (kg/m²), mean (SD)	24.13 (3.33)	24.06 (3.70)	0.10^c^ (62.3)	.92
Skeletal muscle mass (kg), mean (SD)	20.94 (2.64)	21.23 (3.10)	−0.47^c^ (65.6)	.64
Fat-free mass (kg), mean (SD)	39.17 (4.41)	39.30 (5.21)	−0.12^c^ (66.0)	.91
Body fat mass (kg), mean (SD)	20.24 (5.91)	20.13 (7.30)	0.08^c^ (68.8)	.94
Percent body fat (%), mean (SD)	33.51 (5.34)	33.04 (7.27)	0.35^c^ (75.0)	.73
Visceral fat area (cm²), mean (SD)	101.67 (33.42)	98.50 (45.09)	0.38^c^ (74.4)	.70
Waist-to-hip ratio	0.89 (0.04)	0.90 (0.06)	−0.44^c^ (81.0)	.66
Whole-body ECW/TBW^e^, mean (SD)	0.3890 (0.0064)	0.3815 (0.0072)	5.10^c^ (63.0)	<.001
Whole-body 50-kHz phase angle (°), mean (SD)	4.64 (0.66)	5.23 (0.62)	−4.15^c^ (53.4)	<.001

a Continuous variables were compared with 2-sided Welch *t* tests in 95 participants. Tumor laterality was compared with a 2 × 2 Pearson *χ2* test, and procedure type was compared with an omnibus 2 × 3 Pearson *χ*2 test. All 95 participants were included. The *P* values are unadjusted and exploratory. Prognostic nutritional index was calculated as albumin in g/L plus 5 times the absolute lymphocyte count in 10⁹/L.

b NAC: neoadjuvant chemotherapy.

c *t* test.

d Chi-squared test.

e ECW/TBW: extracellular water to total body water ratio.

**Figure 1.**
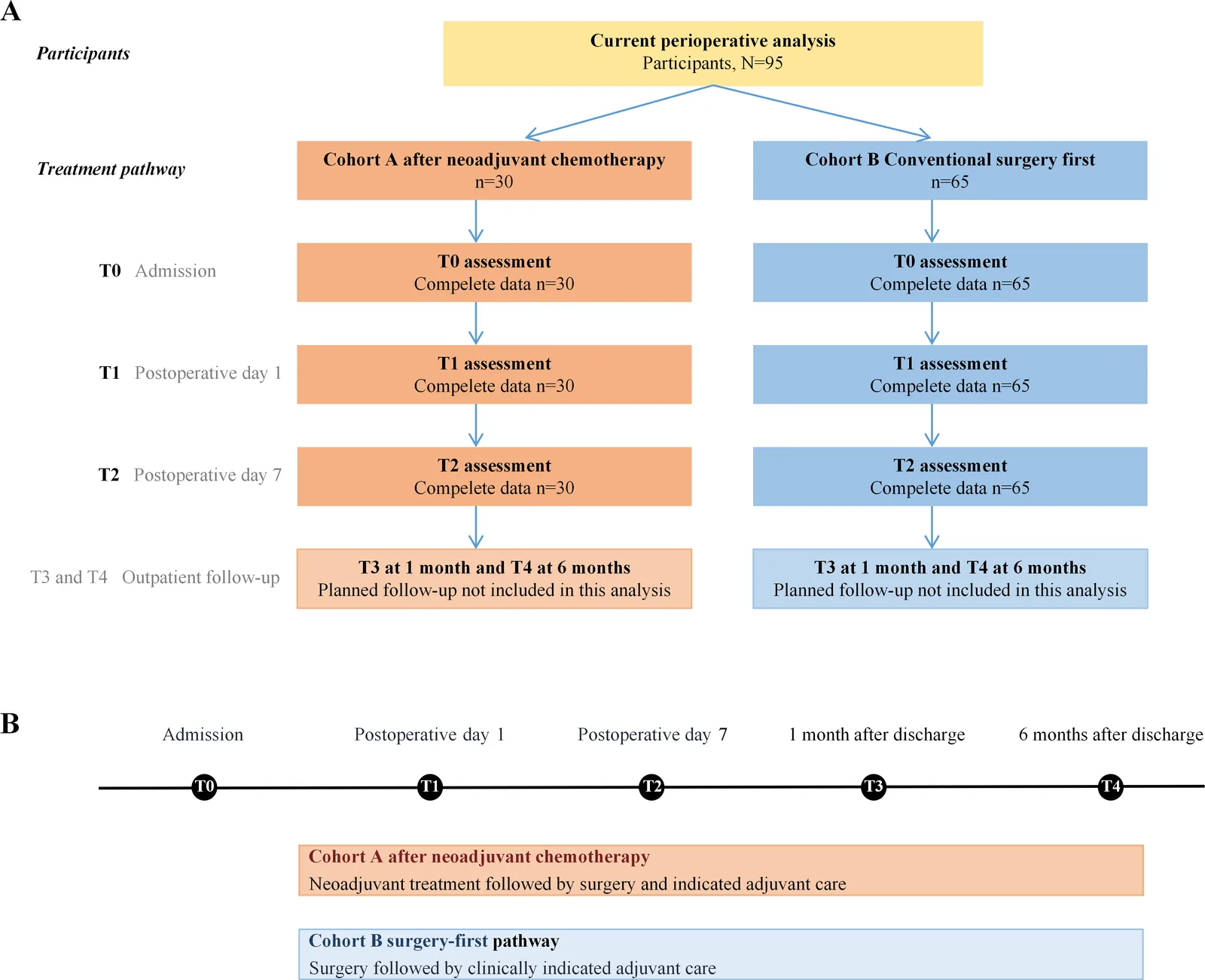
Study cohort and assessment schedule. Panel A summarizes the analysis population and treatment pathways. The analysis included 30 participants in cohort A and 65 in cohort B. Procedure type was available for all 95 participants, who also had complete data for the 13 longitudinal variables at T0, T1, and T2. Panel B shows the assessment schedule. T0 was admission before surgery, T1 was postoperative day 1 after mobilization, and T2 was postoperative day 7. T3 and T4 were scheduled at 1 and 6 months after discharge but were not included in the current analysis.

Only 1 participant in the 95-participant analytic cohort had a discrepancy in the recorded surgical procedure category. On August 3, 2026, this participant’s medical and study records were reviewed. The review confirmed that this participant had undergone modified radical mastectomy, and the procedure-category code was corrected from 4 to 1. The correction affected only procedure classification. No T0, T1, or T2 measurement was changed, and this participant remained in all analyses.

### Characteristics in the T0 Assessment

Mean age was 51.57 (SD 8.28) years in cohort A and 52.03 (SD 12.65) years in cohort B (Table 4). Procedure distribution differed between cohorts (*χ*²_2_=13.76; *P*=.001). Modified radical mastectomy was recorded for 22 of 30 (73.33%) women in cohort A and 22 of 65 (33.85%) in cohort B. Breast-conserving surgery was recorded for 5 of 30 (16.67%) women and 17 of 65 (26.15%) women, respectively. Total mastectomy was recorded for 3 of 30 (10%) women and 26 of 65 women (40%), respectively.

At T0, cohort A minus cohort B mean differences were 5.13 IU/L for aspartate aminotransferase and 8.18 IU/L for alanine aminotransferase. The corresponding 95% CIs were 2.04 to 8.21 and 3.35 to 13.02. Mean differences were −3.09 g/L for total protein and −2.05 g/L for albumin. Their 95% CIs were −5.18 to −1.00 and −3.76 to −0.35. The absolute lymphocyte count difference was −0.63 ×10⁹/L (95% CI −0.83 to −0.42 ×10⁹/L). The prognostic nutritional index difference was −5.19 (95% CI −7.30 to −3.08). This index was calculated from albumin and lymphocyte count. These measures were not used to diagnose malnutrition. The Global Leadership Initiative on Malnutrition framework requires screening and diagnostic assessment with phenotypic and aetiologic criteria [25]. Those criteria were unavailable in the current data cut.

Unadjusted T0 between-cohort *P* values exceeded 0.05 for body weight, BMI, skeletal muscle mass, fat-free mass, and body fat mass. The same applied to percent body fat, visceral fat area, and waist-to-hip ratio (Table 4). Mean body weight was 59.41 (SD 9.34) kg in cohort A and 59.42 (SD 10.58) kg in cohort B. Other cohort means for mass and adiposity measures are reported in Table 4. Whole-body ECW/TBW was higher in cohort A, with a mean difference of 0.0075 (95% CI 0.0046 to 0.0104; *P*<.001). Whole-body 50-kHz phase angle was lower in cohort A, with means of 4.64° (SD 0.66) and 5.23° (SD 0.62). The mean difference was −0.59° (95% CI −0.88° to −0.31°; *t*_53.4_=−4.15; *P*<.001).

### Perioperative Changes Within Cohorts

Each within-cohort 95% CI included zero for weight, BMI, skeletal muscle mass, fat-free mass, body fat mass, percent body fat, and visceral fat area. Each corresponding unadjusted *P* value exceeded 0.05 (Table 2). Mean body weight changed by −0.14 (SD 0.78) kg in cohort A and −0.23 (SD 2.08) kg in cohort B. Changes in skeletal muscle mass were −0.11 and −0.02 kg, respectively. Changes in fat-free mass were −0.14 and +0.08 kg, while body fat mass changed by +0.40 and −0.32 kg. Device-reported skeletal muscle index changed by −0.13 kg/m² in cohort A (95% CI −0.22 to −0.04 kg/m²; *P*=.008). The corresponding change in cohort B was −0.04 kg/m² (95% CI −0.10 to 0.02 kg/m²; *P*=.15). Whole-body ECW/TBW changed little in cohort A and increased by 0.0023 in cohort B (95% CI 0.0011 to 0.0035; *P*<.001).

Mean upper-limb reactance ipsilateral to the recorded tumor side decreased in both cohorts. At 5 kHz, changes were −3.05 Ω in cohort A and −3.78 Ω in cohort B. The corresponding *t* statistics were −6.51 (*df* 29) and −10.50 (*df* 64), with *P*<.001 for both. At 50 kHz, changes were −3.26 and −3.80 Ω. The corresponding *t* statistics were −4.91 (*df* 29) and −8.54 (*df* 64), with *P*<.001 for both.

### Whole-Body and Segmental Phase Angle

Whole-body and segmental phase angles describe different anatomical scales (Figure 2). At T0, mean whole-body 50-kHz phase angle was 4.64° (SD 0.66) in cohort A and 5.23° (SD 0.62) in cohort B. Ipsilateral upper-limb means were 4.18° (SD 0.63) and 4.71° (SD 0.64), respectively. Contralateral upper-limb means were 4.35° (SD 0.59) and 4.76° (SD 0.60). Right lower-limb means were 4.80° (SD 0.86) and 5.60° (SD 0.80). Left lower-limb means were 4.75° (SD 0.79) and 5.46° (SD 0.81). Trunk means did not follow this pattern and were 6.79° (SD 1.00) and 6.68° (SD 0.89). No inferential comparison across anatomical segments was performed. These cross-sectional values were obtained after neoadjuvant chemotherapy in cohort A and before surgery in cohort B. They do not estimate a treatment effect or establish a prognostic threshold.

**Figure 2.**
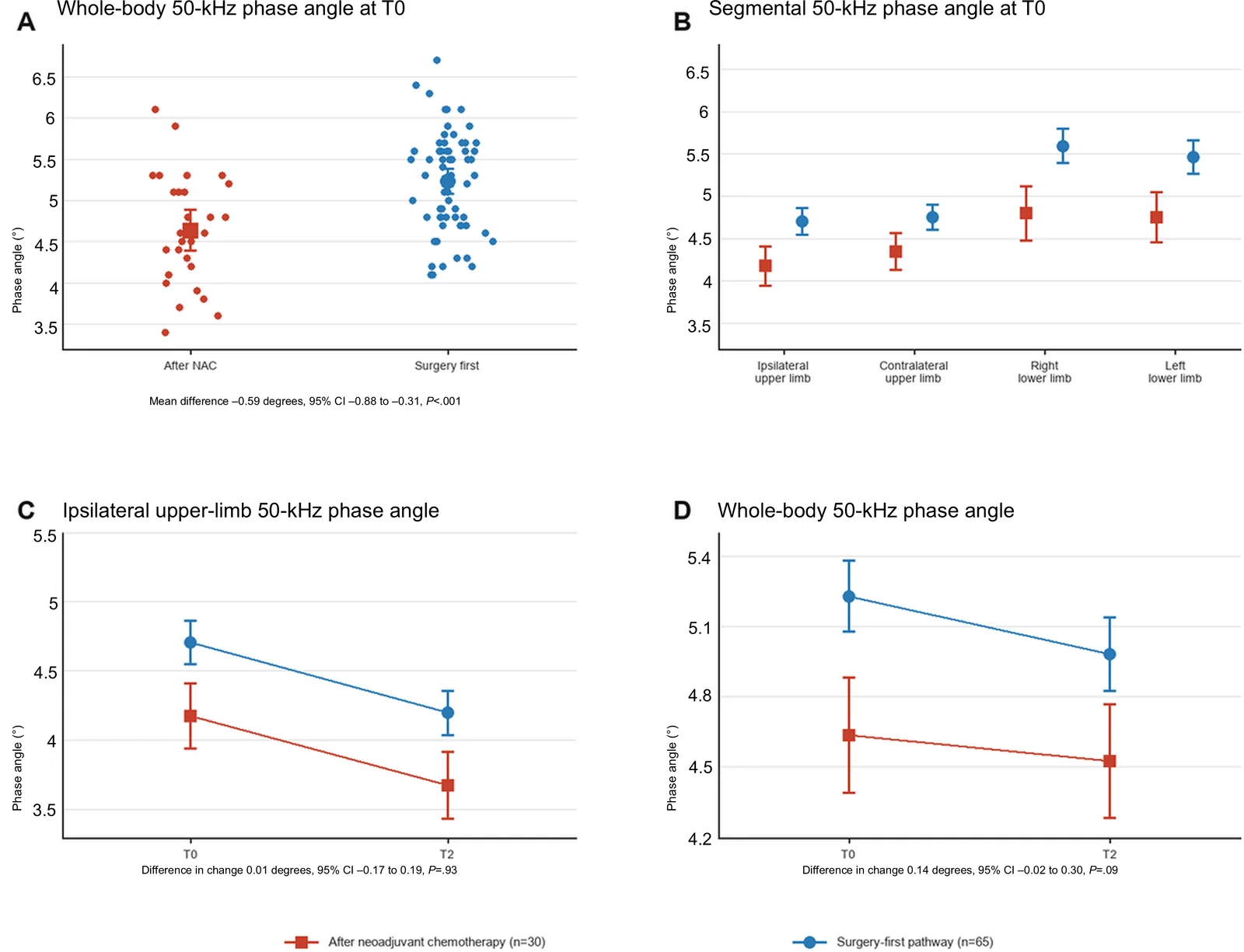
Whole-body and segmental phase-angle findings. Panel A shows whole-body 50-kHz phase angle at T0 for all 95 participants. Panel B shows upper-limb and lower-limb phase angle at T0. Panel C shows ipsilateral upper-limb phase angle at T0 and T2. Panel D shows whole-body phase angle at T0 and T2. Small points in panel A represent individual participants. Large markers represent cohort means. Lines connect cohort means and do not represent individual trajectories. Vertical bars show 95% CIs for cohort means. The annotation in panel A is the T0 mean difference. Annotations in panels C and D are between-cohort differences in mean T0-to-T2 change. All annotated differences are cohort A minus cohort B. The T0 comparison used a 2-sided Welch *t* test. Within-cohort comparisons used 2-sided paired *t* tests. Between-cohort differences in change used 2-sided Welch *t* tests on participant-level change scores. Displayed *P* values are unadjusted. Table 3 reports Benjamini-Hochberg *q* values for the 13 between-cohort change comparisons.

Mean ipsilateral upper-limb 50-kHz phase angle decreased between T0 and T2 in both cohorts. It changed from 4.18° to 3.68° in cohort A and from 4.71° to 4.20° in cohort B. Mean changes were −0.50° (95% CI −0.66° to −0.34°; *P*<.001) and −0.51° (95% CI −0.60° to −0.42°; *P*<.001). The between-cohort difference in mean change was 0.01° (95% CI −0.17° to 0.19°; *P*=.93). Whole-body phase angle changed by −0.11° in cohort A and −0.25° in cohort B. The corresponding between-cohort difference was 0.14° (95% CI −0.02° to 0.30°; *P*=.09). These data do not establish that the upper-limb change reflects lymphatic injury or predicts later lymphoedema.

### Between-Cohort Differences in Mean Change

Mean fat-free mass changed by −0.14 (SD 1.22) kg in cohort A and +0.08 (SD 1.39) kg in cohort B. The between-cohort difference was −0.23 kg (95% CI −0.79 to 0.33 kg; *P*=.42; Table 3 and Figure 3). Mean body fat mass changed by +0.40 (SD 2.58) kg and −0.32 (SD 1.67) kg, respectively. The between-cohort difference was +0.72 kg (95% CI −0.32 to 1.76 kg; *P*=.17). Percent body fat changed by +0.59 and −0.33 percentage points. The difference was +0.93 percentage points (95% CI −0.50 to 2.36; *P*=.20). Visceral fat area increased in cohort A and decreased in cohort B. The difference was +3.79 cm² (95% CI −2.69 to 10.26 cm²; *P*=.25). Device-reported skeletal muscle index changed by −0.13 and −0.04 kg/m². The between-cohort difference was −0.08 kg/m² (95% CI −0.19 to 0.02 kg/m²; *P*=.12). Whole-body ECW/TBW had a between-cohort difference in mean change of −0.0024 (95% CI −0.0045 to −0.0004; *P*=.02; *q*=0.268). None of the 13 between-cohort comparisons had a *q* value less than 0.05.

**Figure 3.**
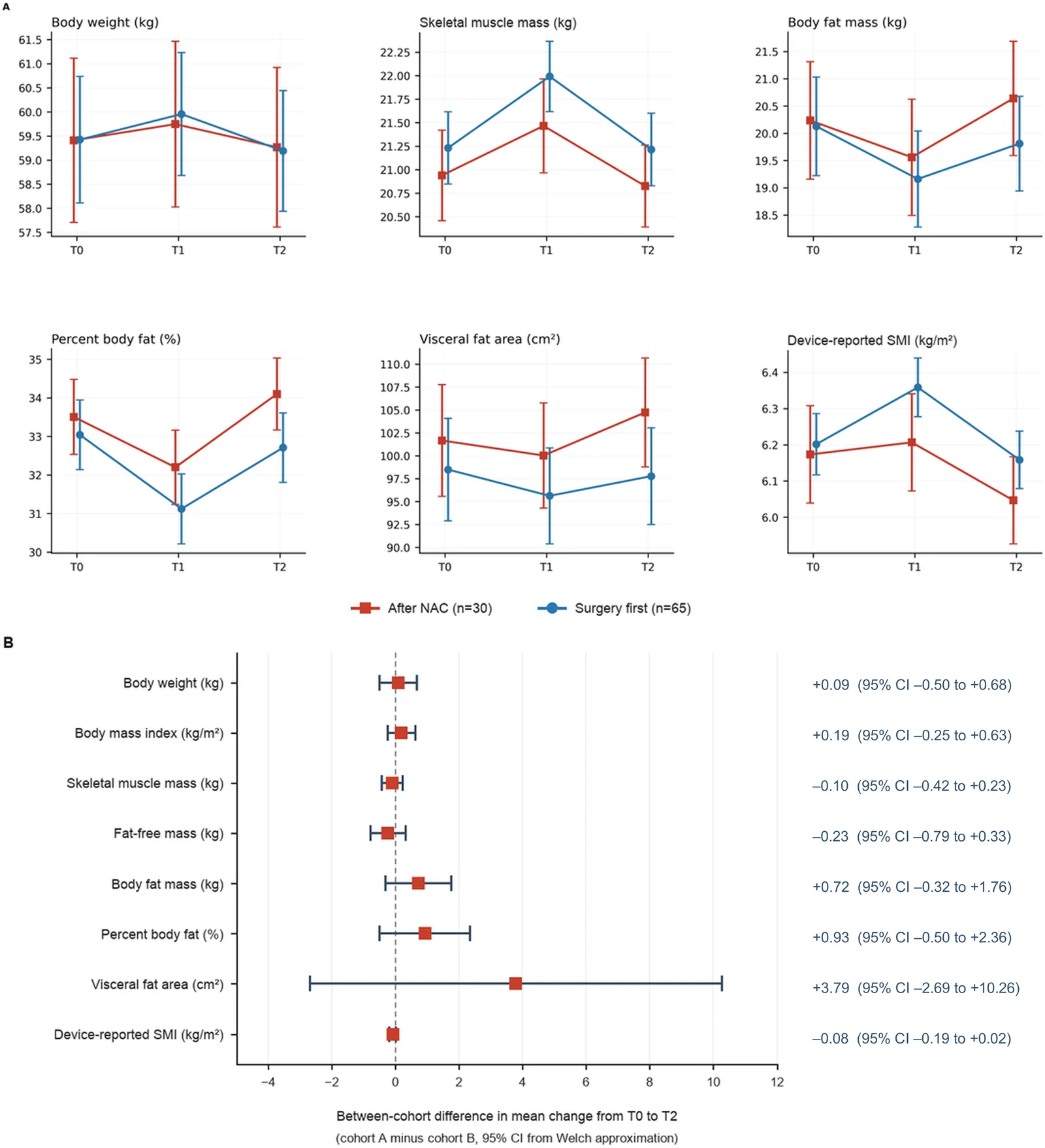
Pilot body-composition results in the T0, T1, and T2 assessments (n=95; cohort A n=30; cohort B n=65). Panel A shows 6 device-reported body-composition measures at T0, T1, and T2. Values are means with SEs for cohort A (n=30) and cohort B (n=65). Square and circular markers show cohort means, connecting lines join the means, and vertical bars show SEs. T1 measurements are descriptive and were excluded from inferential comparisons. Panel B shows cohort A minus cohort B differences in mean T0-to-T2 change for 8 body-composition measures. Squares are point estimates, horizontal bars are 95% CIs from Welch *t* tests, and the vertical reference line marks zero. NAC: neoadjuvant chemotherapy; SMI: device-reported skeletal muscle index.

The post hoc sensitivity analysis included 29 participants in cohort A and 64 in cohort B. Between-cohort differences in body fat mass, percent body fat, and visceral fat area decreased to 0.12 kg, 0.23 percentage points, and 0.22 cm². In cohort A, the whole-body phase-angle estimate changed from −0.11° to −0.15° after the same exclusions. The main estimate had a 95% CI of −0.25 to 0.03 (*P*=.11; post hoc *q*=0.290). The sensitivity estimate had a 95% CI of −0.26 to −0.03 (*P*=.02; post hoc *q*=0.038). The sensitivity estimate for the between-cohort difference was 0.10° (95% CI −0.04° to 0.24°; *P*=.16; *q*=0.643). No *q* value for the 13 between-cohort comparisons was below 0.05 in either analysis.

### Perioperative Assessment Completeness and Feasibility

All 95 participants had complete data for the 13 variables in Table 2 at T0, T1, and T2. All 5 prespecified perioperative feasibility thresholds were met. The preliminary sample-size threshold was exceeded. Retention from T0 to T2 was 95 of 95, and T2 primary-outcome completeness was 100%. Thirteen of 15 whole-body indices met the baseline-balance criterion. Loss to follow-up before T2 was 0 of 95 (Table 5). Screening throughput, assessment burden, interrater reliability, procedure-attributable serious adverse events, and T3/T4 follow-up were not evaluated in the current analysis.

**Table 5.** Prespecified perioperative feasibility metrics and observed performance^a^.

Metric	Prespecified target	Observed in the current data	Status
Preliminary sample size	At least 50 participants	95 participants	Met
Retention from T0 to T2	At least 85%	95/95 (100%)	Met
Completeness of the primary outcome at T2	At least 90%	95/95 (100%)	Met
Baseline balance across 15 whole-body indices	At least 80% with *P* ≥.10	13/15 (87%)	Met
Loss to follow-up before T2	Less than 15%	0/95 (0%)	Met
Eligibility-screening throughput	At least 4 participants per week	Screening log not included	Not evaluated
Acceptability of assessment burden	At least 80% with a rating of 4/10 or lower	Burden ratings and withdrawal reasons not included	Not evaluated
Serious adverse events attributable to study procedures	0	Case-report-form safety data not included	Not evaluated

a The 5 perioperative thresholds were prespecified. Retention and completeness were calculated for all 95 participants across T0, T1, and T2.

## Discussion

### Principal Findings

This exploratory BC-NUTRITION pilot analysis yielded 3 main observations. First, the treatment pathways differed in selected laboratory measures, whole-body ECW/TBW, and whole-body phase angle at T0. These contrasts involved biochemical and bioelectrical measures rather than most BIA-derived mass estimates. The treatment pathway was observational, cohort A lacked a prechemotherapy assessment, and relevant clinical covariates were incomplete. The T0 differences, therefore, cannot be attributed to neoadjuvant chemotherapy. Second, ipsilateral upper-limb reactance and phase angle decreased in both cohorts between T0 and T2. Evidence for a between-cohort difference in phase-angle change was limited. Third, none of the 13 between-cohort change comparisons had a *q* value less than 0.05. All 5 prespecified perioperative feasibility thresholds were met between T0 and T2. Assessment acceptability and outpatient retention were not evaluated.

The T0 whole-body phase-angle difference is clinically interesting because it co-occurred with lower albumin and absolute lymphocyte count and higher ECW/TBW in cohort A. However, phase angle is an indirect bioelectrical measure influenced by tissue composition, hydration, measurement frequency, and acquisition conditions [10,26]. The cross-sectional contrast may reflect treatment history, disease characteristics, treatment-pathway selection, fluid distribution, or other unmeasured factors. It does not establish a chemotherapy-induced decrement, and the present data do not support applying a prognostic cut-off derived from other populations with cancer. Similarly, the prognostic nutritional index was a derived laboratory index and cannot substitute for a diagnostic malnutrition assessment [25]. These T0 findings are best treated as hypotheses for prospective evaluation with a prechemotherapy baseline and standardized measurement conditions.

The ipsilateral upper-limb signal concerns a different anatomical scale. Its similar decrease in both cohorts suggests a common perioperative phenomenon, but the mechanism cannot be identified from the available data. Potential contributors include local surgical effects, perioperative fluid shifts, inflammation, positioning, the type and extent of axillary management, and measurement variability. Previous breast cancer studies evaluated upper-limb impedance and interlimb ratios for lymphedema detection [13,14]; they did not validate short-term segmental phase-angle change as a direct marker of lymphatic injury. Because axillary procedure, perioperative intravenous fluid exposure, measurement timestamps, and later edema outcomes were unavailable, the present upper-limb change neither demonstrates lymphatic injury nor predicts subsequent lymphedema. Whole-body and segmental findings should therefore be interpreted separately.

The multiplicity-adjusted results are central to interpretation. The unadjusted between-cohort change in whole-body ECW/TBW had a *P* value of .02, but its *q* value was 0.268. No between-cohort comparison had a *q* value less than 0.05. CIs for several clinically relevant differences were wide and included zero. These findings do not establish equivalence between pathways. The pilot was not designed or powered as an equivalence study. Excluding 2 participants attenuated several body-fat estimates and changed the cohort A whole-body phase-angle result. This sensitivity illustrates the influence of potentially discordant measurements. Future analyses should use prespecified data-quality rules and verify unexpected values against original measurement documentation.

### Comparison With Prior Work

Prior studies provide context for these patterns. One longitudinal computed-tomography (CT) study reported decreased CT-derived skeletal muscle index and increased visceral fat index during neoadjuvant chemotherapy [15]. Other studies examined pretreatment body composition in relation to treatment response or survival [9,16]. A prospective observational protocol also included longitudinal body-composition assessment during chemotherapy [17]. These studies did not report a contemporaneous comparison of perioperative change between post-neoadjuvant and surgery-first cohorts. The present between-cohort difference in body-fat-mass change was +0.72 kg (95% CI −0.32 to 1.76 kg). However, follow-up began after neoadjuvant chemotherapy in cohort A, so the estimate does not represent change during chemotherapy.

Phase-angle studies in breast cancer vary substantially in population, measurement method, and outcome definition, and evidence for survival associations is inconsistent [11]. Associations reported in populations with advanced cancer may not generalize to a perioperative cohort with predominantly operable disease [12]. The current study adds a contemporaneous treatment-pathway comparison and both whole-body and segmental measures, but its exploratory design and incomplete clinical covariates preclude causal or prognostic conclusions.

### Limitations

Strengths include the prospective parent-study framework, a contemporaneous surgery-first comparison, and whole-body and segmental bioimpedance measurements. Data for all selected measures were complete at T0, T1, and T2 for all 95 participants. The analysis reports CIs, multiplicity-adjusted *q* values, and a transparent sensitivity analysis. It also separates cross-sectional T0 contrasts from perioperative changes and distinguishes whole-body from segmental phase angles.

Several limitations remain. Treatment pathway was observed rather than assigned, creating confounding by indication. The absence of a prechemotherapy assessment prevents estimation of change during neoadjuvant chemotherapy. Procedure type was imbalanced, axillary management was not separately recorded, and stage, subtype, regimen, toxicity, and perioperative fluid data were incomplete. Screening throughput, assessment-burden ratings, interrater reliability, procedure-attributable serious adverse events, participant-level timestamps, and T3/T4 follow-up data were unavailable. Single-center recruitment and eligibility restrictions limit generalizability. BIA-derived body-composition estimates are not equivalent to CT-derived or dual energy X-ray absorptiometry (DXA)–derived measures and depend on predictive equations and acquisition procedures [26]. Measurements were standardized with an InBody 770, but participant-level timestamps and a reference-method comparison were unavailable. Short-term BIA-derived changes should therefore not be interpreted as direct tissue gain or loss. The *q*-value adjustment and 2-participant sensitivity analysis were post hoc. The pilot was not designed for confirmatory or equivalence testing.

Procedure distribution differed markedly between cohorts, with modified radical mastectomy recorded in 73.33% (22/30) of cohort A and 33.85% (22/65) of cohort B. Because the dataset did not separately capture axillary procedure or extent, this imbalance limits the interpretation of ipsilateral bioimpedance. Tumor stage, molecular subtype, chemotherapy regimen, timing and cumulative exposure, treatment toxicity, and perioperative fluid administration were also insufficiently characterized for adjusted or regimen-specific analyses. Future parent-study analyses should prospectively capture these variables, distinguish breast from axillary procedures, and define an adjustment strategy before outcome analysis.

Methodological priorities are equally important. A prechemotherapy baseline is needed to separate treatment-pathway selection from within-person change during neoadjuvant therapy. Future analyses should retain the standardized InBody 770 procedure and record participant-level measurement timestamps and relevant clinical covariates. A reference-method substudy using CT or DXA would help characterize the performance of BIA-derived mass estimates [26]. Completion of T3 and T4 follow-up is required to determine whether perioperative measures are reproducible; recover over time; or associate with later nutritional, functional, or quality-of-life outcomes.

### Conclusions

This exploratory pilot analysis identified cross-sectional differences in selected laboratory and whole-body bioelectrical measures between women presenting for surgery after neoadjuvant chemotherapy and women in a surgery-first pathway. Mean upper-limb phase angle ipsilateral to the recorded tumor side decreased in both pathways between T0 and T2. No between-cohort change comparison remained significant after Benjamini-Hochberg adjustment. Whole-body and segmental findings should be interpreted separately, and their clinical relevance remains uncertain. All 5 prespecified perioperative feasibility thresholds were met within T0 to T2. Assessment acceptability, procedure-attributable safety, and outpatient retention require dedicated case-report forms and T3/T4 follow-up. Future work should include a prechemotherapy baseline, the standardized InBody 770 procedure, participant-level timestamps, relevant clinical covariates, prospective data-quality checks, and T3/T4 follow-up.

The present findings support retaining phase angle as an exploratory repeated measure within the parent study, but not yet as a clinical stratification threshold or surrogate endpoint. The most informative next step is prospective validation across a prechemotherapy baseline, the perioperative period, and T3/T4 follow-up under standardized acquisition, with relevant clinical covariates and prespecified analysis. Only after reproducibility, prognostic relevance, and clinically meaningful change have been established should phase-angle-guided nutrition, exercise, or lymphedema-prevention interventions be evaluated.

## Supplementary material

10.2196/102885 Multimedia Appendix 1 Bounded parent-study framework, completeness of perioperative data, and post hoc sensitivity analyses

10.2196/102885 Checklist 1 STROBE checklist

## Abbreviations

BC-NUTRITION: Body Composition and Nutrition trajectories in breast cancer
BIA: bioelectrical impedance analysis
CT: computed tomography
DXA: dual energy X-ray absorptiometry
ECW/TBW: extracellular water to total body water ratio
FACT-B: Functional Assessment of Cancer Therapy-Breast
NRS-2002: Nutritional Risk Screening 2002
PASS: Power Analysis and Sample Size
PG-SGA: Patient-Generated Subjective Global Assessment
PHQ-9: Patient Health Questionnaire-9
