# Supplementary material for: Perioperative Body-Composition and Phase-Angle Measures in Women Undergoing Breast Cancer Surgery After Neoadjuvant Chemotherapy or Treated Through a Surgery-First Pathway (BC-NUTRITION): Exploratory 2-Cohort Pilot Analysis
Source: JMIR Form Res. 2026 Sep 30;10:e102885. doi: 10.2196/102885 (PMC13625174; doi:10.2196/102885)
Supplement: Supplementary file 2 [file formative-v10-e102885-s001.docx]

***Multimedia Appendix 1***

**Perioperative body-composition and phase-angle measures in women undergoing breast cancer surgery after neoadjuvant chemotherapy or through a surgery-first pathway (BC-NUTRITION), an exploratory two-cohort pilot analysis**

*Supplementary methods and sensitivity analyses*

**Contents**

Appendix S1. Bounded parent-study framework

Appendix S2. Completeness of perioperative data

Appendix S3. Post hoc sensitivity analyses

# **Appendix S1. Bounded parent-study framework**

The main manuscript reports the exploratory 95-participant perioperative pilot. Table S1 summarizes the parent-study elements that are relevant to interpreting how the pilot can inform later work. These elements were not used to support the pilot estimates.

## **Table S1. Concise parent-study framework**

| **Element** | **Bounded parent-study plan** |
| --- | --- |
| Sample-size context | The parent target is 214 participants, with 107 per cohort, based on the assumptions stated in Methods. The pilot SDs provide preliminary variance estimates. Any endpoint-specific update will also require a clinically important difference and the within-participant correlation. |
| Longitudinal analysis | Each definitive analysis will prespecify the endpoint, analysis population, cohort-by-time contrast, covariates, multiplicity family, and missing-data method. Repeated measures will be analysed at participant level, with effect estimates and 95% confidence intervals. |
| Missing data | The extent and reasons for missingness will be summarized by assessment. Primary models and sensitivity analyses will state their assumptions and the number of participants contributing to each estimate. |
| Causal framework | Clinical and temporal knowledge will guide covariate selection. Observational comparisons will remain interpreted as associations rather than causal effects. |
| Exploratory prediction modelling | Prediction modelling will proceed only if T3 and T4 follow-up, outcome events, and complete assessment sequences support the candidate predictor set. Development and validation will report calibration, discrimination, and limits on clinical use. |
| Monitoring arrangements | Arrangements for monitoring recruitment, data completeness, measurement quality, and safety will be documented before the definitive analysis. |
| Patient involvement | Future patient involvement may inform participant materials, outcome priorities, visit burden, and dissemination. Patient involvement was not evaluated as an outcome in the present pilot. |
| Equity and generalisability | The definitive study will describe recruitment, follow-up, and outcome completeness across relevant demographic and socioeconomic groups when the available data and subgroup sizes permit. |

*Note. The parent-study framework is separated from the pilot methods and results. No definitive longitudinal, causal, or prediction model was fitted in the present analysis. SD = standard deviation.*

# **Appendix S2. Completeness of perioperative data**

Bioelectrical impedance variables were obtained with the InBody 770. The reported analyses used measurements at 5 kHz and 50 kHz and followed the procedure described in Methods. Table S2 reports completeness for the variables used in the T0 to T2 analyses and for the InBody Score, which was not analysed.

## **Table S2. Completeness of selected perioperative variables**

| **Variable** | **T0, n/N** | **T1, n/N** | **T2, n/N** |
| --- | --- | --- | --- |
| Body weight, kg | 95/95 | 95/95 | 95/95 |
| Body mass index, kg/m² | 95/95 | 95/95 | 95/95 |
| Skeletal muscle mass, kg | 95/95 | 95/95 | 95/95 |
| Fat-free mass, kg | 95/95 | 95/95 | 95/95 |
| Body fat mass, kg | 95/95 | 95/95 | 95/95 |
| Percent body fat, % | 95/95 | 95/95 | 95/95 |
| Visceral fat area, cm² | 95/95 | 95/95 | 95/95 |
| Device-reported skeletal muscle index, kg/m² | 95/95 | 95/95 | 95/95 |
| Whole-body ECW/TBW | 95/95 | 95/95 | 95/95 |
| Whole-body 50-kHz phase angle, ° | 95/95 | 95/95 | 95/95 |
| Ipsilateral upper-limb 5-kHz reactance, Ω | 95/95 | 95/95 | 95/95 |
| Ipsilateral upper-limb 50-kHz reactance, Ω | 95/95 | 95/95 | 95/95 |
| Ipsilateral upper-limb 50-kHz phase angle, ° | 95/95 | 95/95 | 95/95 |
| InBody Score, not analysed | 93/95 | 90/95 | 93/95 |

*Note. The 13 variables used in the T0 to T2 analyses were complete for all 95 participants. InBody Score was excluded from analysis and was missing for 2 participants at T0, 5 at T1, and 2 at T2. T0 was admission before surgery, T1 was postoperative day 1 after mobilisation, and T2 was postoperative day 7. ECW/TBW = extracellular water to total body water ratio.*

# **Appendix S3. Post hoc sensitivity analyses**

The main analysis retained all 95 participants. The sensitivity analysis omitted two participants who met data-quality audit flags defined during revision. One flag was an absolute discrepancy greater than 3 kg/m² between recorded T2 body mass index and body mass index recalculated from T2 weight using height inferred from T0. The other was an absolute T0 to T2 weight change greater than 10 kg. The same two participants were omitted from every sensitivity comparison, and the recorded values were unchanged.

## **Table S3. Between-cohort differences in mean change**

| **Variable** | **Main analysis Cohort A n=30, cohort B n=65** | **Sensitivity analysis Cohort A n=29, cohort B n=64** |
| --- | --- | --- |
| Body weight, kg | +0.09 (−0.50, +0.68); p = 0.760; q = 0.823 | −0.17 (−0.56, +0.22); p = 0.396; q = 0.643 |
| Body mass index, kg/m² | +0.19 (−0.25, +0.63); p = 0.391; q = 0.607 | −0.08 (−0.24, +0.08); p = 0.301; q = 0.643 |
| Skeletal muscle mass, kg | −0.10 (−0.42, +0.23); p = 0.551; q = 0.652 | −0.15 (−0.47, +0.17); p = 0.360; q = 0.643 |
| Fat-free mass, kg | −0.23 (−0.79, +0.33); p = 0.420; q = 0.607 | −0.29 (−0.86, +0.28); p = 0.307; q = 0.643 |
| Body fat mass, kg | +0.72 (−0.32, +1.76); p = 0.169; q = 0.455 | +0.12 (−0.36, +0.61); p = 0.606; q = 0.740 |
| Percent body fat, % | +0.93 (−0.50, +2.36); p = 0.198; q = 0.455 | +0.23 (−0.60, +1.05); p = 0.581; q = 0.740 |
| Visceral fat area, cm² | +3.79 (−2.69, +10.26); p = 0.245; q = 0.455 | +0.22 (−3.33, +3.77); p = 0.903; q = 0.903 |
| Device-reported skeletal muscle index, kg/m² | −0.08 (−0.19, +0.02); p = 0.121; q = 0.455 | −0.12 (−0.21, −0.02); p = 0.019; q = 0.225 |
| Whole-body ECW/TBW | −0.0024 (−0.0045, −0.0004); p = 0.021; q = 0.268 | −0.0021 (−0.0040, −0.0002); p = 0.035; q = 0.225 |
| Whole-body 50-kHz phase angle, ° | +0.14 (−0.02, +0.30); p = 0.092; q = 0.455 | +0.10 (−0.04, +0.24); p = 0.159; q = 0.643 |
| Ipsilateral upper-limb 5-kHz reactance, Ω | +0.72 (−0.46, +1.90); p = 0.226; q = 0.455 | +0.50 (−0.61, +1.61); p = 0.369; q = 0.643 |
| Ipsilateral upper-limb 50-kHz reactance, Ω | +0.54 (−1.06, +2.14); p = 0.502; q = 0.652 | +0.30 (−1.20, +1.81); p = 0.689; q = 0.747 |
| Ipsilateral upper-limb 50-kHz phase angle, ° | +0.01 (−0.17, +0.19); p = 0.931; q = 0.931 | −0.04 (−0.19, +0.12); p = 0.626; q = 0.740 |

*Note.* Values are cohort A minus cohort B differences in mean T2 minus T0 change with 95% confidence intervals. Two-sided p values were obtained from Welch two-sample t tests. The q values use Benjamini-Hochberg adjustment across the 13 comparisons within each analysis.

## **Table S4. Within-cohort changes**

| **Variable** | **Cohort** | **Main analysis** | **Sensitivity analysis** |
| --- | --- | --- | --- |
| Body weight, kg | A | −0.14 (−0.43, +0.15); p = 0.318; q = 0.547 | −0.18 (−0.47, +0.10); p = 0.203; q = 0.452 |
| Body weight, kg | B | −0.23 (−0.75, +0.28); p = 0.370; q = 0.547 | −0.01 (−0.29, +0.26); p = 0.920; q = 0.945 |
| Body mass index, kg/m² | A | +0.11 (−0.28, +0.50); p = 0.569; q = 0.668 | −0.07 (−0.19, +0.05); p = 0.226; q = 0.452 |
| Body mass index, kg/m² | B | −0.08 (−0.29, +0.13); p = 0.455; q = 0.591 | +0.01 (−0.10, +0.12); p = 0.842; q = 0.945 |
| Skeletal muscle mass, kg | A | −0.11 (−0.38, +0.16); p = 0.397; q = 0.547 | −0.14 (−0.41, +0.14); p = 0.312; q = 0.541 |
| Skeletal muscle mass, kg | B | −0.02 (−0.20, +0.17); p = 0.855; q = 0.889 | +0.01 (−0.17, +0.19); p = 0.903; q = 0.945 |
| Fat-free mass, kg | A | −0.14 (−0.60, +0.31); p = 0.525; q = 0.650 | −0.16 (−0.63, +0.31); p = 0.496; q = 0.717 |
| Fat-free mass, kg | B | +0.08 (−0.26, +0.43); p = 0.623; q = 0.675 | +0.13 (−0.20, +0.47); p = 0.423; q = 0.647 |
| Body fat mass, kg | A | +0.40 (−0.56, +1.37); p = 0.400; q = 0.547 | −0.02 (−0.45, +0.40); p = 0.908; q = 0.945 |
| Body fat mass, kg | B | −0.32 (−0.73, +0.10); p = 0.129; q = 0.305 | −0.15 (−0.39, +0.09); p = 0.221; q = 0.452 |
| Percent body fat, % | A | +0.59 (−0.76, +1.95); p = 0.377; q = 0.547 | +0.02 (−0.69, +0.74); p = 0.945; q = 0.945 |
| Percent body fat, % | B | −0.33 (−0.83, +0.16); p = 0.183; q = 0.367 | −0.20 (−0.63, +0.22); p = 0.346; q = 0.562 |
| Visceral fat area, cm² | A | +3.07 (−2.91, +9.04); p = 0.302; q = 0.547 | +0.57 (−2.64, +3.78); p = 0.719; q = 0.890 |
| Visceral fat area, cm² | B | −0.72 (−3.38, +1.94); p = 0.591; q = 0.668 | +0.35 (−1.25, +1.96); p = 0.662; q = 0.890 |
| Device-reported skeletal muscle index, kg/m² | A | −0.13 (−0.22, −0.04); p = 0.008; q = 0.022 | −0.15 (−0.23, −0.07); p < 0.001; q = 0.003 |
| Device-reported skeletal muscle index, kg/m² | B | −0.04 (−0.10, +0.02); p = 0.148; q = 0.321 | −0.03 (−0.09, +0.02); p = 0.258; q = 0.478 |
| Whole-body ECW/TBW | A | −0.0001 (−0.0018, +0.0016); p = 0.903; q = 0.903 | +0.0003 (−0.0013, +0.0018); p = 0.716; q = 0.890 |
| Whole-body ECW/TBW | B | +0.0023 (+0.0011, +0.0035); p < 0.001; q = 0.001 | +0.0024 (+0.0011, +0.0036); p < 0.001; q < 0.001 |
| Whole-body 50-kHz phase angle, ° | A | −0.11 (−0.25, +0.03); p = 0.112; q = 0.290 | −0.15 (−0.26, −0.03); p = 0.015; q = 0.038 |
| Whole-body 50-kHz phase angle, ° | B | −0.25 (−0.33, −0.16); p < 0.001; q < 0.001 | −0.25 (−0.34, −0.16); p < 0.001; q < 0.001 |
| Ipsilateral upper-limb 5-kHz reactance, Ω | A | −3.05 (−4.01, −2.09); p < 0.001; q < 0.001 | −3.29 (−4.15, −2.43); p < 0.001; q < 0.001 |
| Ipsilateral upper-limb 5-kHz reactance, Ω | B | −3.78 (−4.49, −3.06); p < 0.001; q < 0.001 | −3.79 (−4.52, −3.06); p < 0.001; q < 0.001 |
| Ipsilateral upper-limb 50-kHz reactance, Ω | A | −3.26 (−4.62, −1.90); p < 0.001; q < 0.001 | −3.57 (−4.82, −2.33); p < 0.001; q < 0.001 |
| Ipsilateral upper-limb 50-kHz reactance, Ω | B | −3.80 (−4.69, −2.91); p < 0.001; q < 0.001 | −3.88 (−4.77, −2.98); p < 0.001; q < 0.001 |
| Ipsilateral upper-limb 50-kHz phase angle, ° | A | −0.50 (−0.66, −0.34); p < 0.001; q < 0.001 | −0.55 (−0.67, −0.42); p < 0.001; q < 0.001 |
| Ipsilateral upper-limb 50-kHz phase angle, ° | B | −0.51 (−0.60, −0.42); p < 0.001; q < 0.001 | −0.51 (−0.60, −0.42); p < 0.001; q < 0.001 |

*Note.* Values are participant-level mean T2 minus T0 changes with 95% confidence intervals. Two-sided p values were obtained from paired t tests. The post hoc q values were calculated separately across the 26 comparisons in the main analysis and the 26 comparisons in the sensitivity analysis.
